# Eye Movements and Cognitive Strategy in a Fluid Intelligence Test: Item Type Analysis

**DOI:** 10.3389/fpsyg.2018.00380

**Published:** 2018-03-21

**Authors:** Paulo G. Laurence, Tatiana P. Mecca, Alexandre Serpa, Romain Martin, Elizeu C. Macedo

**Affiliations:** ^1^Social and Cognitive Neuroscience Laboratory and Developmental Disorders Program, Center for Health and Biological Sciences, Mackenzie Presbyterian University, São Paulo, Brazil; ^2^Educational Psychology Post-Graduation Program, Centro Universitário FIEO, Osasco, Brazil; ^3^Hogrefe CETEPP, São Paulo, Brazil; ^4^Faculty of Language and Literature, Humanities, Arts and Education, University of Luxembourg, Luxembourg City, Luxembourg

**Keywords:** intelligence, eye tracking, cognition, logical reasoning, problem solving

## Abstract

Eye movements help to infer the cognitive strategy that a person uses in fluid intelligence tests. However, intelligence tests demand different relations/rules tokens to be solved, such as rule direction, which is the continuation, variation or overlay of geometric figures in the matrix of the intelligence test. The aim of this study was to understand whether eye movements could predict the outcome of an intelligence test and in the rule item groups. Furthermore, we sought to identify which measure is best for predicting intelligence test scores and to understand if the rule item groups use the same strategy. Accordingly, 34 adults completed a computerized intelligence test with an eye-tracking device. The toggling rate, that is, the number of toggles on each test item equalized by the item latency explained 45% of the variance of the test scores and a significant amount of the rule tokens item groups. The regression analyses also indicated toggling rate as the best measure for predicting the score and that all the rule tokens seem to respect the same strategy. No correlation or difference were found between baseline pupil size and fluid intelligence. *Wiener Matrizen-Test 2* was demonstrated to be a good instrument for the purpose of this study. Finally, the implications of these findings for an understanding of cognition are discussed.

## Introduction

Research has shown that psychophysiological measures can be employed as predictors of fluid intelligence (Schlagenhauf et al., 2013; Finn et al., 2015; Friedal et al., 2015), including aspects of ocular movement analysis such as pupillometry (Hayes and Petrov, 2016), scanpath (Hayes et al., 2015) and strategy (Vigneau et al., 2006). Fluid intelligence (Gf) refers to problem-solving capacities as well as identifying relationships and drawing inferences under novel conditions, especially those that do not rely on or require the minimal amount of previously acquired knowledge; that is, when there are not enough schemes, habits and knowledge to respond to demands. In essence, Gf is the ability to solve new problems through reasoning (Horn and Blankson, 2012; Schneider and McGrew, 2012).

Eye movement analysis during Gf tasks has helped in understanding problem-solving (Thibaut and French, 2016; Vendetti et al., 2017). This type of data is very beneficial for understanding the strategies individuals employ to solve a task and identify the best strategy (Bethell-Fox et al., 1984; Hayes et al., 2015). The strategy can be inferred though eye movements. According to previous studies, eye gaze behavior can reflect a cognitive strategy (Snow, 1980; Bethell-Fox et al., 1984).

Bethell-Fox et al. (1984) suggested that two types of strategies exist in analogy tasks with multiple alternatives: constructive matching and response elimination. The strategy of constructive matching involves solving the exercise first and then moving on to the alternatives. On the contrary, response elimination involves making multiple comparisons between the alternatives and the analogy question in an attempt to eliminate the answers that do not fit the model. In their study, Bethell-Fox et al. (1984) aimed to explore the differences of the individuals in the performance of a geometric analogies task. To obtain these data, 28 subjects took a geometric analogies task (i.e., A is to B as C is to “?”) with multiple alternatives by utilizing a computer equipped with eye-tracking. They analyzed the fixation count and the fixation sequence between the three boxes of the analogy question and the alternatives. In the eye-tracking analysis, it was expected that the strategy of response elimination would have larger amounts of alternations/re-inspections between the analogy question and the alternatives than the constructive matching strategy. These data were satisfactory when revealing the distinction between the strategies by means of eye-tracking; however, the data was limited because of the eye-tracking technology of the time. Their results suggested that constructive matching is a better strategy than response elimination.

Vigneau et al. (2006) tested these strategies for matrix intelligence tests and, as expected, obtained the same results. Their objective was to understand, with the help of eye-tracking, the cause of individual differences in a matrix-based intelligence test. In their study of Vigneau et al. (2006), developed three groups of eye-tracking measures; refer to the measures in the discussion of the method. The three groups were as follows: measures of alternation between interest areas (an alteration of the measures of Bethell-Fox et al., 1984); time on interest areas; and a measure of time distribution in the matrix cells, which is referred to as the Matrix Time Distribution Index (MTDI). In intelligence tests, these alternations/re-inspections between the matrix and the alternatives were called toggles. The authors also commented that with these measures it would be possible to replicate the findings of Bethell-Fox et al. (1984) concerning the strategies. However, time on matrix-based intelligence tests instead of analogy tasks demonstrated more toggles would indicate a bigger reliance on response elimination strategy and fewer toggles would suggest a constructive matching strategy (Vigneau et al., 2006). The results also suggested that MTDI, a measure of matrix inspection, would be the best predictor for high scores in the matrix-based intelligence test.

**FIGURE 1:**
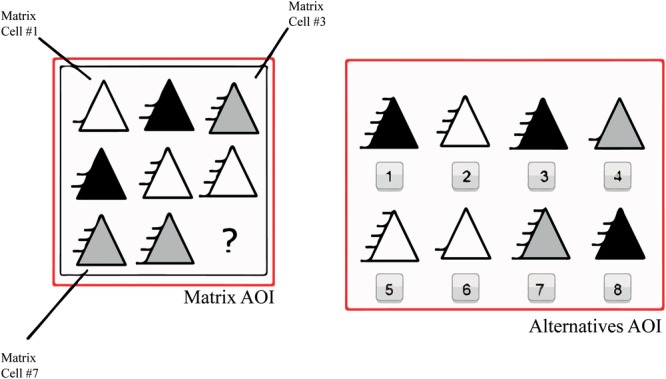
WMT-2 example (rule direction group) and the Areas of Interest (AOIs). Matrix AOIs are divided into 8 cells; 1 is on the top left, 3 is on the top right and 8 on the bottom right.

Although there are several matrix intelligence tests, Raven’s Advanced Progressive Matrices (RAPM) has been employed in the majority of studies (Vigneau et al., 2006; Hayes et al., 2011; Vakil and Lifshitz-Zehavi, 2012). RAPM has been most often used to infer problem-solving strategies from the analysis of eye movements. This may be explained by the fact that this test is more common than others (Kaplan and Saccuzzo, 2008). However, RAPM may not be the best test for analyzing eye movements because the test was not developed with a consideration for rules, even though there are signs that RAPM follows various rules (Carpenter et al., 1990). Rules, in matrix-based intelligence tests, can be defined as distinct relations that must be made by the person so as to reach the correct answer (Carpenter et al., 1990) An example of a rule is the relation of an element that will increase or decrease in each column or row; an example thereof is the lines to the left of the triangles, which are portrayed in **Figure 1**.

Rules can be an excellent way of arriving at a superior understanding of the strategies that a person employs to solve a test question. Furthermore, tests designed based on rules can be better than RAPM for eye-tracking studies since there is better control of the intervenient variables. One test that was developed by taking rules into consideration is the *Wiener Matrizen-Test 2* (WMT-2). WMT-2 is a matrix-based Gf test. It has a 3 × 3 matrix with the ninth cell missing and eight possible alternatives on the right. Each item of the test has a rule that subjects the subject have to to complete the matrix. WMT-2 was designed to evaluate three tokens: *rule type*, which is the continuation, variation or overlay of geometric figures; *rule direction*, which is the variation in direction of geometric figures, whether horizontal, vertical or a combination of both; and *graphic component nature*, which is the variation in shape, number, pattern or spatial arrangement of the graphic elements of geometric figures (Schlottfeldt and Malloy-Diniz, 2014).

One advantage of WMT-2 arises from the combination of the approaches of the Rasch Model and linear logistic test model during the item selection phase in test development. This enables the developer to add those cognitive operations for solving items to the total item difficulties. This rationality addresses item resolution so as to understand cognitive abilities and thus, facilitates description and hypothesizing regarding the schemas and strategies utilized by respondents (Formann and Piswanger, 1979; Reif, 2012). These properties are valid for both paper-and-pencil and computerized versions of WMT-2, making it a better measure for computer protocols (Formann et al., 2011; Malloy-Diniz and Schlottfeldt, in press). At present, several studies have employed WMT-2 as a Gf measure (e.g., Hergovich and Arendasy, 2005; Flegr et al., 2012), especially in German-speaking countries (Ingold et al., 2015).

The aim of this study was to understand whether eye movements and strategy can predict the outcome of an intelligence test and in particular, in the rule tokens item groups. Attempts were also made to identify which measure is more superior for predicting intelligence test scores and to understand if the rules item groups respect the same strategy or if there are any differences.

## Materials and Methods

### Subjects

A total of 34 university students (20 women) were recruited from Mackenzie Presbyterian University in São Paulo, Brazil to participate in the study. Their ages (*M* = 21.10, *SD* = 2.15) ranged from 18 to 28 years, and they had normal or corrected-to-normal vision.

### Stimuli

Participants were shown 21 trials of WMT-2, namely, three practice examples and 18 real test questions as well as the test instructions. WMT-2 has a 3 × 3 matrix with eight possible answers on the right (see **Figure 1**). Inside of Matrix AOI, there are 9 cells; cells 1–8 have the figures with the variations/rules and the ninth cell is empty. Items 1, 2, 3, and 5 represent the rule type item group; items 4, 6, 7, 9, and 10 depict the rule direction item group; and item 8 along with items 11–18 portray the graphic component nature item group (Schlottfeldt and Malloy-Diniz, 2014). The computerized version of the test was employed. Each test page had 1366 × 786 pixels. Before the test instructions, a black fixation point was displayed for 6 s against a gray background, and between each trial, the fixation point was displayed for 3 s.

### Apparatus

The device utilized for the tests was a RED500 eye-tracking device from SensoMotoric Instruments (2014; sampling rate 500 Hz). WMT-2 was set in SMI Experiment Center^TM^ version 3.7.104. All data on the eyes were collected. However, only the data for the right eye was used because the data from both eyes are usually positively correlated. Furthermore, the use of data from one eye facilitates the analysis (Glynn and Rosner, 2012; Karakosta et al., 2012; Armstrong, 2013). For 10.89% of the duration of the task, the participants were not looking at the computer monitor or if they were, the eye-tracking device was not able to identify this. Thus, there was a loss of 10.89% of the data. The registration errors were interpolated according to the standard parameters of the SMI BeGaze^TM^ version 3.7.104. In addition, the calibration and validation were conducted by following the standard procedures for SMI iView^TM^. The eye movement parameters were set as the default in SMI BeGaze^TM^ version 3.7.104.

### Procedure

This study was carried out in accordance with the recommendations of the Ethics Committee of Research of the Mackenzie Presbyterian University. All subjects gave written informed consent. The protocol was approved by the Ethics committee under CAAE number 75035917.5.0000.0084. Subjects were placed in a room and seated approximately 70 cm away from a 22” computer monitor that was equipped with the eye-tracking device. The room had constant lighting during the whole procedure; the average room luminance was 687 cd/m^2^. After the subjects were seated, calibration and validation were completed by following the standard procedure for SMI iView^TM^ with 9-points. The instructions for WMT-2 were first displayed; this was followed by the first three trials as practice examples, which did not count toward the score. After the examples, subjects proceeded with the test. Each time the subjects selected an answer, they said the number of the item aloud. The researcher then pressed the button to proceed to the next trial.

### Eye-Tracking Measures

The following variables were measured: item latency, which is the sum of the time spent on each item from its appearance until pressing the key; time on matrix, that is, the time spent on the cells of the matrix in seconds; time on alternatives, namely, time spent on answer options in seconds; proportional time on matrix and on alternatives, which is the time spent on each one of the categories divided by item latency; number of toggles, namely, the number of times the subject alternated from matrix to alternatives and vice versa; rate of toggling, which is defined as the number of toggles divided by item latency, with high rates indicating less time between each toggle; latency to first toggle, that is, time spent on the trial before the first toggle in seconds; and the MTDI, which is calculated by the sum of the time spent on cells 1, 2, 4 ,and 5 min the sum of time spent on cells 3, 6, 7, 8, and 9, with negative values indicating that observation was predominant on the last column and last row. These variables were employed by Vigneau et al. (2006) and Vakil and Lifshitz-Zehavi (2012), but were adapted for WMT-2 (see **Figure 1**). These measures were derived from the eye gaze of the participants thought the marking of the Areas of Interest (AOIs) over the matrix and the alternatives (see **Figure 1**). An AOI is a selected area, which is possible to analyze separately from the rest of the stimulus. With these AOIs, it was possible to calculate the time on the matrix and alternatives. The number of toggles was acquired each time the person’s gaze moved from matrix AOI to alternatives AOI or vice versa. The MTDI was calculated from the time spent in each matrix cell AOI, obtained in the SMI BeGaze^TM^. The item latency was timed by the SMI BeGaze^TM^. All other variables were calculated through these measures. Also, pupil size was measured in the first fixation point to access the baseline pupil size. The baseline pupil size was calculated as the average pupil diameter (mm) only when the subject was fixating.

### Data Analysis

Two indexes were employed to conduct the internal consistency analysis. Firstly, Guttman’s Lambda-2 coefficient was utilized because research has indicated that it is a better estimate of reliability, especially when the sample is small such as in the present study (Sijtsma, 2012). Secondly, the split-half method with an adjustment by Spearman-Brown coefficient as a measure of homogeneity of the items was employed. We divided WMT-2 between the even and odd items. We also conducted a normality test for the WMT-2 score.

We performed descriptive statistics of each measure for the full test and for every rule item group. We also used Pearson correlation coefficient to determine if there was an association between the eye movements, the pupil size and the test score. Also, to replicate the Tsukahara et al. (2016) findings with the baseline pupil size and Gf, we calculated the tertile scores of WMT-2 and used an independent sample *t*-test to compare the pupil diameter between the lower tertile and the higher tertile.

Finally, we performed multiple linear regression analyses. A stepwise model with all the eye movement measures as independent variables and the WMT-2 score as the dependent variable was conducted to examine the association of these variables with the test score. The same procedure was conducted for each rule item group measure. Because the Rule Type Group measures did not generate a model, we also performed linear regression analyses (backward method) with the measures of Rule Type Group. Furthermore, we followed the same models of Vigneau et al. (2006) to understand if equivalent associations were found. The first model had a MTDI and proportional time on matrix as independent variables and the second one had a MTDI and latency to first toggle as independent variables. Both employed the full test score as the dependent variable. We used the most appropriate model for the rule item groups measures to see if this association was found in the rule tokens or not.

## Results

### Full Test Results

The analyses to obtain the WMT-2 scores showed adequate values of Guttman’s Lambda-2 (0.70) and the Spearman-Brown Coefficient (0.78). The correlation between the odd and even items was moderate (0.64). These findings indicate good reliability of the WMT-2 scores for this sample.

The descriptive statistics of the variables are displayed in **Table 1**. The distribution of WMT-2 score was normal (skewness = 0.44, kurtosis = -1.025, Shapiro-Wilk test of normality = 0.141, n.s.). There was a positive, significant correlation of large magnitude between the task time and score in WMT-2 (*r* = 0.57, *p <* 0.001). The subjects did not look at the matrix and alternatives AOI approximately 12% of the time. Individuals toggled one time in 3.155 s on average, and there was a significant negative correlation of large magnitude between the rate of toggling and WMT-2 scores (*r* = -0.68, *p <* 0.001). The latency to the first toggle showed a positive, significant correlation of large magnitude with the WMT-2 score (*r* = 0.55, *p* = 0.001). The MTDI of the subjects did not correlate with the individuals’ scores (*r* = 0.21, p = 0.232, n.s.). There was no significant correlation between the WMT-2 score and the baseline pupil diameter (*r* = -0.22, *p* = 0.216). The baseline pupil diameter mean was 3.52 mm (*SD* = 0.43). When comparing the baseline pupil size between the lower tertile (*M* = 3.62 mm, *SD* = 0.49) and the higher tertile (*M* = 3.61 mm, *SD* = 0.37) of WMT-2, there was no significant difference, *t*(24) = 0.80, p = 0.434.

**Table 1:** Descriptive statistics of WMT-2 measures.

				Groups		
	Full test			RT	RD	GCN
		
	Mean (*SD*)	Min.	Max	Mean (*SD*)		
Percent correct	58.5 (17.3)	27.8	88.9	88.9 (20.5)	61.7 (24.3)	43.1 (21.3)
Item latency (seconds)	39.820 (17.732)	8.829	86.043	21.598 (10.501)	34.853 (14.550)	50.679 (25.407)
Time on matrix (seconds)	27.081 (12.869)	5.682	56.379	15.402 (8.404)	23.671 (10.655)	35.072 (18.948)
Proportional time on matrix	0.68 (0.06)	0.50	0.77	0.68 (0.06)	0.66 (0.06)	0.67 (0.06)
Time on alternatives (seconds)	7.938 (2.867)	2.213	13.672	4.016 (1.867)	7.890 (3.334)	9.708 (3.987)
Proportional time on alternatives	0.22 (0.04)	0.14	0.28	0.21 (0.04)	0.23 (0.05)	0.20 (0.04)
Number of toggles	10.63 (3.68)	3.44	18.66	6.99 (4.12)	10.60 (3.31)	12.25 (5.16)
Rate of toggling	0.317 (0.092)	0.170	0.556	0.361 (0.113)	0.336 (0.100)	0.287 (0.111)
Latency to first toggle (seconds)	10.99 (5.78)	2.79	24.81	8.09 (5.66)	9.29 (5.24)	13.23 (8.11)
Matrix time distribution index	0.041 (0.167)	–0.229	0.494	0.072 (0.175)	–0.044 (0.192)	0.079 (0.181)

### Rule Item Groups Results

The group means are presented in **Table 1**. The rule tokens item group with the highest score was the Rule Type Group, with 88.9%, and the group with the lowest score was the Graphic Component Nature Group, with 43.1%. As expected, the Graphic Component Nature Group had more item latency and the Rule Type Group had less item latency. Time on the matrix showed the same pattern as item latency, with the Rule Type Group demonstrating less time on the matrix. The same pattern occurred in time on the alternatives, with the Graphic Component Nature Group having more time on the alternatives. Proportional time on the matrix did not show significant variation; the amount was approximately 4%. Similarly, the proportional time on the alternatives varied by only 3%. The number of toggles followed the same pattern as item latency, with Rule Type having the fewest toggles. The Rule Type Group toggled once every 2.77 s in comparison to the the Rule Direction Group and Graphic Component Nature Group who toggled every 2.98 and 3.48 s, respectively. Thus, the rate of toggling varied by 0.074. Latency to the first toggle, as expected, was greater in the Graphic Component Nature Group and less in the Rule Type Group. Finally, the MTDI varied from -0.044 (Rule Direction Group) to 0.079 (Graphic Component Nature Group).

### Predicting Test Score Based on the Measures

To examine the association of these variables with the WMT-2 score, multiple linear regression analyses were conducted. The correlation of the variables is displayed in **Table 2**. First, a stepwise model was conducted with all the variables as independent variables and test score as the dependent variable. This generated one model (multiple *R* = 0.68), with the rate of toggling as the predictor. The rate of toggling could predict 45% of the variation in the full test score. This predictor had a negative correlation with the test score. The coefficients are presented in **Table 3**.

**Table 2:** Correlations between WMT-2 score, item latency and the eye movement measures.

	WMT-2	IL	ToM	PToM	ToA	PToA	NT	RoT	LFT	MTDI
WMT-2 score	1.00	0.52^∗∗^	0.55^∗∗^	0.26	0.28	–0.58^∗∗^	0.10	–0.68^∗∗^	0.55^∗∗^	0.22
Item latency		1.00	0.98^∗∗^	0.44^∗∗^	0.83^∗∗^	–0.58^∗∗^	0.66^∗∗^	–0.64^∗∗^	0.70^∗∗^	0.26
Time on matrix			1.00	0.58^∗∗^	0.78^∗∗^	–0.62^∗∗^	0.65^∗∗^	–0.63^∗∗^	0.69^∗∗^	0.28
Proportional time on matrix				1.00	0.25	–0.51^∗∗^	0.38^∗^	–0.22	0.28	0.24
Time on alternatives					1.00	–0.09	0.78^∗∗^	–0.42^∗^	0.53^∗∗^	0.02
Proportional time on alternatives						1.00	–0.13	0.59^∗∗^	–0.48^∗∗^	–0.37^∗∗^
Number of toggles							1.00	0.02	0.18	0.08
Rate of toggling								1.00	–0.715^∗∗^	–0.09
Latency to first toggle									1.00	0.07
Matrix time distribution index										1.00

*^∗^*p* < 0.05; ^∗∗^*p* < 0.01.*

**Table 3:** Regression models and coefficients for WMT-2 full test and each rule token item group.

	Beta	*t*	Sig.	Correlation coefficients			Tolerance
				Zero-order	Partial	Semi-partial	
**Full test model (*R*^2^ = 0.46, adjusted *R*^2^ = 0.45)**							
Rate of toggling	–0.681	–5.261	<0.001	–0.681	–0.681	–0.681	1.000
**Group RT model (*R*^2^ = 0.52, adjusted *R*^2^ = 0.39)**							
Item latency	8.832	2.771	0.010	0.302	0.477	0.376	0.002
Time on matrix	–7.919	–2.808	0.009	0.290	–0.482	–0.381	0.002
Percentage time on matrix	1.430	2.739	0.011	0.134	0.473	0.371	0.067
Time on alternatives	–2.699	–3.448	0.002	0.183	–0.560	–0.467	0.030
Percentage time on alternatives	1.450	3.302	0.003	–0.135	0.544	0.448	0.095
Number of toggles	1.581	2.874	0.008	0.140	0.491	0.390	0.061
Rate of toggling	–1.281	–3.717	0.001	–0.337	–0.589	–0.504	0.155
**Group RD model (*R*^2^ = 0.25, adjusted *R*^2^ = 0.22)**							
Rate of toggling	–0.496	–3.230	0.003	–0.496	–0.496	–0.496	1.000
**Group GCN model (*R*^2^ = 0.32, adjusted *R*^2^ = 0.29)**							
Rate of toggling	–0.561	–3.833	0.001	–0.561	–0.561	–0.561	1.000

The same process was conducted for the rule tokens item groups, with the percentage of correct answers for each group serving as the dependent variable. The Rule Type Group did not generate a model by the stepwise method; therefore, a backward method was conducted and this generated a significant model (multiple *R* = 0.72) with the following predictors: rate of toggling, proportional time on alternative, time on alternative, proportional time on matrix, time on matrix and number of toggles. These predictors could predict approximately 39% of the Rule Type Group score. The Rule Direction Group generated one model (multiple *R* = 0.50) with the rate of toggling as the predictor; this predictor explained 22% of the variance of the Rule Direction group score. The Graphic Component Nature Group had one model (multiple *R* = 0.56); the rate of toggling was the predictor. The rate of toggling could predict 29% of the variation of the Graphic Component Nature Group score. The coefficients are also shown in **Table 3**.

We employed the same models as Vigneau et al. (2006) to determine whether the same associations were found. To do this, two linear regression analyses were conducted with total test score as the dependent variable: Model 1 of Vigneau et al., with the MTDI and proportional time on matrix as independent variables (multiple *R* = 0.30, *R*^2^ = 0.09, adjusted *R*^2^ = 0.03), and Model 2 of Vigneau et al., with the MTDI and latency to first toggle as independent variables (multiple *R* = 0.58, *R*^2^ = 0.34, adjusted *R*^2^ = 0.29).

Finally, the most appropriate model (Vigneau et al.’s second model) was used to analyze the rule tokens item groups, but with the percentage of correct answers for each group as the dependent variable: for Rule Type Group (*R*^2^ = 0.08, adjusted *R*^2^ = 0.02), multiple *R* = 0.28; for Rule Direction Group (*R*^2^ = 0.21, adjusted *R*^2^ = 0.16) multiple *R* = 0.46; and for Graphic Component Nature Group (*R*^2^ = 0.32, adjusted *R*^2^ = 0.27), multiple *R* = 0.56. This implies that the model best predicted the variation of Graphic Component Nature Group, followed by Rule Direction Group and barely predicts the Rule Type Group.

## Discussion

The objective of this study was to investigate whether eye movements and strategy could predict the outcome of the WMT-2 test in the rule tokens item groups as well as to identify which measure is better for predicting scores and whether the rules tokens groups respect the same strategy. For this purpose, we adopted the measures employed by Vigneau et al. (2006) and Vakil and Lifshitz-Zehavi (2012).

The mean percentage of correct answers was as expected based on previous studies such as those conducted by Vigneau et al. (2006), Hayes et al. (2011), and Vakil and Lifshitz-Zehavi (2012) in the typical development group. However, the average item latency was lower in comparison with other studies. Unlike previous studies, WMT-2 was employed instead of RAPM as a measure of Gf, and this could explain the difference. Although they measure the same concept, the nature of the tests is different. The rate of toggling was as expected in comparison to Vigneau et al.’s (2006) study.

The WMT-2 has its rules clearly defined by the items, which is beneficial for analyzing intelligence in different levels of intellectual performance (Schlottfeldt and Malloy-Diniz, 2014). The groups were different in various respects. Graphic Component Nature was more difficult than the others; eye-movement measures were influenced by that. On the other hand, Rule Type was much easier and thus, we observed lower item latency, number of toggles, rate of toggling and latency to first toggle. With respect to the level of difficulty, Rule Direction was situated somewhere between Rule Type and Graphic Component Nature. Although Graphic Component Nature had more items, Rule Direction appeared to be a better predictor of test and eye-movements outcomes; this is shown in **Table 1**. Except for the MTDI and proportional times, all of the measures for the Rule Direction Group were the closest to the general results. Therefore, Rule Direction may be the best predictor of test results.

In relation to the baseline pupil size, we find an average size smaller than that of Tsukahara et al. (2016). This was expected since our experiment was conducted under higher luminance conditions. Also, our fixation point to access the baseline pupil size was of 6 s and their fixation point was of 30 s. With that said, our results showed no difference between the pupil diameter and the Gf of the participants. Additionally, no correlation was found between the baseline pupil size and the intelligence test score. In fact, the tendency was a negative correlation. Unsworth and Robison (2017) also could not replicate Tsukahara et al. (2016) findings. A possible explanation would be that this phenomenon is only observable under lower luminance conditions. Coyne et al. (2017) also conducted a similar experiment under higher luminance conditions and did not obtain significant correlations, but their tendency was a positive correlation.

In all three rule tokens item groups as well as in the test score regression analysis, the rate of toggling emerged as a fundamental measure. The regression analysis explained approximately 45% of total score: this is similar to that of the models of Vigneau et al. (2006) and Hayes et al. (2011). The rate of toggling may be defined as the number of toggles standardized by time. This is an important measure for predicting strategy (Bethell-Fox et al., 1984; Vigneau et al., 2006). Individuals that utilize a constructive matching strategy should make fewer comparisons and have a lower rate of toggling, while individuals who employ response elimination strategy should carry out more toggles and have a higher rate thereof. With our regression models, as expected, the rate of toggling proved to be the best measure for distinguishing strategy, and predicting test outcomes and rule tokens results. This measure also indicated, through the Beta coefficient, that fewer comparisons should mean more right answers. Furthermore, it is important to emphasize that this measure was found in the three rule tokens required in this test and thus, indicates that it is a more suitable variable for rule research as well as a more credible and universal measure. In addition, all rule tokens item groups respect the same strategy once the rate of toggling emerged in the three groups as the significant predictor and with a negative Beta. This indicated that fewer comparisons equate to more points on the intelligence test.

The MTDI helps one to understand whether subjects tend to focus their analysis in the last row and column. Those that limit their analysis to this part of the matrix should make more errors (Vigneau et al., 2006). In our study, the MTDI displayed a poor correlation with the percentage of correct answers; this differs from the findings of Vigneau et al. (2006). It is possible that this index may not fit WMT-2 matrix logic. The MTDI was employed by only a few studies outlined in the literature review; however, Hayes et al. (2011) used this index and found poor predictability for RAPM score (*R*^2^ = 0.02). Therefore, the MTDI may not be the best variable for predicting test outcomes, but rather may serve as a qualitative tool for understanding eye movements.

When the regression models by Vigneau et al. (2006) were performed, the first did not fit with our study (*R*^2^ = 0.09). However, when performed for the rule tokens item groups, for the second model, although it showed the same adjusted *R*^2^ = 0.29 as Vigneau et al., only the Graphic Component Nature Group had a similar adjusted R^2^. Consequently, this may imply that this model does cannot predict WMT-2 results, but rather, only for Graphic Component Nature scores. Our regression model showed the adjusted *R*^2^ = 0.29, a result that was close to that in their model.

Is important to note that WMT-2 is a computerized test (Malloy-Diniz and Schlottfeldt, in press) while RAPM is a virtual adapted version of the paper-and-pencil test. However, the full WMT-2 test was utilized for this study while other studies have generally employed a short version of RAPM that consists of only 14–28 items out of a total of 36. In this study, we were able to use the full test; the sessions took less time than those of studies that used the short version of RAPM. We strongly encourage the use of WMT-2 in studies with computerized intelligence tests.

These results are important in demonstrating the difference between the participants when performing the test. They show how intelligent subjects can allocate their attention in a more efficient way. The subjects with better Gf scores could better divide their time in each one of the AOIs and find the relevant variations of the test. The lack of toggle between the matrix and alternatives indicate a better process of investigating the relevant information in each AOI and then making the decision. It is possible that subjects have an optimal time to identify the variation patterns in the matrix and then go to the alternatives. One may also infer that the strategies are related to working memory. The constructive matching strategy should allow them to gather more information and hold onto it until the decision regarding the alternative has been made. The response elimination strategy may be linked to low working memory and thus, the subject would have to move between the matrix and the alternatives to retain the information. The correlation of working memory and fluid intelligence is not a novel concept (see Engle et al., 1999; Conway et al., 2003). Rather, working memory is a fluid intelligence predictor (Conway et al., 2002). Furthermore, the difficulty of fluid intelligence tests is correlated with working memory because the harder the task is, the more information the subject has to retain to find the correct answer (Little et al., 2014).

In relation to the data, eye-tracking is a good method for understanding the strategies because it is possible to infer the strategy from the data collected. Techniques like electroencephalography or functional magnetic resonance imaging still cannot give you direct access to the strategy the person is employing although various studies have investigated this (see Liang et al., 2014). Questionnaires about the strategy that the subject used can also be employed in this type of study. However, questionnaires are less trustworthy and the sort of data that is collected was not appropriate for the type of analysis that we wanted to conduct.

We opted always to present the stimuli in the same way, that is, with the matrix on the left side and the alternatives on the right so as preserve the characteristics of the test. Although not usual, other studies should mirror the stimuli to see if the results are not because of the position of the stimulus. In other words, other studies should look for position effects in eye-tracking studies when dealing with matrix-based tests. Our study did not have a measure of working memory. With respect to the hypotheses of the present study, we highly recommend the use of working memory measures in studies related to strategies in matrices tests. Furthermore, it is important to note that the study had 34 participants; all of whom were university students. Therefore, it is recommended that the study should be replicated in larger and diverse populations. In addition, with the importance of strategies for solving problems being highlighted and considering human cognitive development, a study with children to demonstrate at what point of the development they begin to utilize both strategies, equal to those used by adults, is necessary. Studies that differentiate the application of these strategies between men and women can also be beneficial so as to understand the characteristics of the use of strategies of resolution. Furthermore, new methods are emerging as tools so as to have a more superior understanding of individual differences in intelligence (Hayes and Petrov, 2016) and for understanding test strategies (Hayes et al., 2011). These should be employed in future studies in this area.

To conclude, it is possible to predict approximately 45% of test results with eye movements and a significant amount between rule tokens item groups. In addition, the rate of toggling was shown to be the most reliable measure for predicting scores in this study. Finally, it was revealed that all the rule token item groups respect the same strategy, as proposed by Bethell-Fox et al. (1984) and Vigneau et al. (2006) with regard to constructive matching strategy. In other words, fewer comparisons equal more right answers.

## Author Contributions

All authors listed have made a substantial, direct and intellectual contribution to the work, and approved it for publication.

## Conflict of Interest Statement

The authors are reporting that AS is an employee of and EM has published a book published with Hogrefe CETEPP, a company that may be affected by the research reported in the enclosed paper. We have disclosed those interests fully to Frontiers. The other authors declare that the research was conducted in the absence of any commercial or financial relationships that could be construed as a potential conflict of interest.

## References

[B1] Armstrong R. A. (2013). Statistical guidelines for the analysis of data obtained from one or both eyes. *Ophthalmic Physiol. Opt.* 33 7–14. 10.1111/opo.12009 23252852

[B2] Bethell-Fox C. E. Lohman D. F. Snow R. E. (1984). Adaptive reasoning: componential and eye movement analysis of geometric analogy performance. *Intelligence* 8 205–238. 10.1016/0160-2896(84)90009-6

[B3] Carpenter P. A. Just M. A. Shell P. (1990). What one intelligence test measures: a theoretical account of the processing in the Raven Progressive Matrices test. *Psychol. Rev.* 97 404–431. 10.1037/0033-295X.97.3.404 2381998

[B4] Conway A. R. Cowan N. Bunting M. F. Therriault D. J. Minkoff S. R. (2002). A latent variable analysis of working memory capacity, short-term memory capacity, processing speed, and general fluid intelligence. *Intelligence* 30 163–183. 10.1016/s0160-2896(01)00096-4

[B5] Conway A. R. Kane M. J. Engle R. W. (2003). Working memory capacity and its relation to general intelligence. *Trends Cogn. Sci.* 7 547–552. 10.1016/j.tics.2003.10.005 14643371

[B6] Coyne J. T. Foroughi C. Sibley C. (2017). Pupil diameter and performance in a supervisory control task: a measure of effort or individual differences? *Proc. Hum. Fact. Ergon. Soc. Annu. Meet.* 61 865–869. 10.1177/1541931213601689

[B7] Engle R. W. Tuholski S. W. Laughlin J. E. Conway A. R. (1999). Working memory, short-term memory, and general fluid intelligence: a latent-variable approach. *J. Exp. Psychol.* 128 309–331. 10.1037//0096-3445.128.3.309 10513398

[B8] Finn E. S. Shen X. Scheinost D. Rosenberg M. D. Huang J. Chun M. M. (2015). Functional connectome fingerprinting: identifying individuals using patterns of brain connectivity. *Nat. Neurosci.* 18 1664–1671. 10.1038/nn.4135 26457551 PMC5008686

[B9] Flegr J. Geryk J. Volný J. Klose J. Černochová D. (2012). Rhesus factor modulation of effects of smoking and age on psychomotor performance, intelligence, personality profile, and health in Czech soldiers. *PLoS One* 7:e49478. 10.1371/journal.pone.0049478 23209579 PMC3509049

[B10] Formann A. K. Piswanger K. (1979). *Wienet Matrizen-Test (WMT) [Viennese Matrices Test]*. Weinheim: Beltz.

[B11] Formann A. K. Waldherr K. Piswanger K. (2011). *Wiener Matrizen-Test 2 (WMT-2): Ein Rasch-Skalierter Sprachfreier Kurztest zur Erfassung der Intelligenz [Viennese Matrices Test 2 (WMT-2): A Rapid-Scaled, Language-Free Short-Circuit Test for the Assesment of Intelligence]*. Göttingen: Hogrefe.

[B12] Friedal E. Schlagenhauf F. Beck A. Dolan R. J. Huys Q. J. Rapp M. A. (2015). The effects of life stress and neural learning signals on fluid intelligence. *Eur. Arch. Psychiatry Clin. Neurosci.* 265 35–43. 10.1007/s00406-014-0519-3 25142177 PMC4311068

[B13] Glynn R. J. Rosner B. (2012). Regression methods when the eye is the unit of analysis. *Ophthalmic Epidemiol.* 19 159–165. 10.3109/09286586.2012.674614 22568429 PMC3454458

[B14] Hayes T. R. Petrov A. A. (2016). Pupil diameter tracks the exploration-exploitation trade-off during analogical reasoning and explains individual differences in fluid intelligence. *J. Cogn. Neurosci.* 28 308–318. 10.1162/jocn_a_00895 26488587

[B15] Hayes T. R. Petrov A. A. Sederberg P. B. (2011). A novel method for analyzing sequential eye movements reveals strategic influence on Raven’s Advanced Progressive Matrices. *J. Vis.* 11:10. 10.1167/11.10.10 21926182

[B16] Hayes T. R. Petrov A. A. Sederberg P. B. (2015). Do we really become smarter when our fluid-intelligence test scores improve? *Intelligence* 48 1–14. 10.1016/j.intell.2014.10.005 25395695 PMC4226176

[B17] Hergovich A. Arendasy M. (2005). Critical thinking ability and belief in the paranormal. *Pers. Individ. Diff.* 38 1805–1812. 10.1016/j.paid.2004.11.008

[B18] Horn J. L. Blankson A. N. (2012). “Foundations for better understanding of cognitive abilities,” in *Contemporary Intellectual Assessment: Theories, Tests and Issues*, 3rd Edn, eds Flanagan D. P. Harrison P. L. (New York, NY: The Guilford Press), 73–98.

[B19] Ingold P. V. Kleinmann M. König C. J. Melchers K. G. (2015). Transparency of assessment centers: lower criterion-related validity but greater opportunity to perform? *Pers. Psychol.* 69 467–497. 10.1111/peps.12105

[B20] Kaplan R. M. Saccuzzo D. P. (2008). *Psychological Testing: Principles, Applications, and Issues*, 7th Edn. Boston, MA: Cengage Learning.

[B21] Karakosta A. Vassilaki M. Plainis S. Elfadl N. H. Tsilimbaris M. Moschandreas J. (2012). Choice of analytic approach for eye-specific outcomes: one eye or two? *Am. J. Ophthalmol.* 153 781–782. 10.1016/j.ajo.2012.01.004 22078901

[B22] Liang P. Jia X. Taatgen N. A. Zhong N. Li K. (2014). Different strategies in solving series completion inductive reasoning problems: an fMRI and computational study. *Int. J. Psychophysiol.* 93 253–260. 10.1016/j.ijpsycho.2014.05.006 24841995

[B23] Little D. R. Lewandowsky S. Craig S. (2014). Working memory capacity and fluid abilities: the more difficult the item, the more more is better. *Front. Psychol.* 5:239. 10.3389/fpsyg.2014.00239 24711798 PMC3968765

[B24] Malloy-Diniz L. Schlottfeldt C. G. (in press). Teste Matrizes de Vienna 2: Versão Informatizada [Vienesse Matrices Test 2: Informatized Version]. São Paulo: Editora Hogrefe Cetepp.

[B25] Reif M. (2012). Applying a construction rational to a rule based designed questionnaire using the Rasch model and LLTM. *Psychol. Test Assess. Model.* 54 73–79.

[B26] Schlagenhauf F. Rapp M. A. Huys Q. J. Beck A. Wüstenberg T. Deserno L. (2013). Ventral striatal prediction error signaling is associated with dopamine synthesis capacity and fluid intelligence. *Hum. Brain Mapp.* 34 1490–1499. 10.1002/hbm.22000 22344813 PMC3731774

[B27] Schlottfeldt C. G. Malloy-Diniz L. (2014). *Teste Matrizes de Vienna 2: Teste de Inteligência não verbal Escalonado Segundo o Modelo Rasch. [Viennese Matrices Test 2: Non-verbal intelligence test staged according to the Rasch model]*. São Paulo: Editora Hogrefe Cetepp.

[B28] Schneider W. J. McGrew K. S. (2012). “The Cattell-Horn-Carroll model of intelligence,” in *Contemporary Intellectual Assessment: Theories, Tests and Issues* 3rd Edn eds Flanagan D. P. Harrison P. L. (New York, NY: The Guilford Press) 99–144.

[B29] SensoMotoric Instruments (2014). *iView Manual (Version 3.4)*. Teltow: SensoMotoric Instruments.

[B30] Sijtsma K. (2012). Future of psychometrics: ask what psychometrics can do for psychology. *Psychometrika* 77 4–20. 10.1007/S11336-011-9242-4

[B31] Snow R. E. (1980). “Aptitude processes,” in *Aptitude, Learning, and Instruction: Cognitive Process Analyses of Aptitude* Vol. 1 eds Snow R. E. Federico P.-A. Montague W. E. (Hillsdale, NJ: Erlbaum) 27–63.

[B32] Thibaut J. P. French R. M. (2016). Analogical reasoning, control and executive functions: a developmental investigation with eye-tracking. *Cogn. Dev.* 38 10–26. 10.1016/j.cogdev.2015.12.002

[B33] Tsukahara J. S. Harrison T. L. Engle R. W. (2016). The relationship between baseline pupil size and intelligence. *Cognit. Psychol.* 91 109–123. 10.1016/j.cogpsych.2016.10.001 27821254

[B34] Unsworth N. Robison M. K. (2017). A locus coeruleus-norepinephrine account of individual differences in working memory capacity and attention control. *Psychon. Bull. Rev.* 24 1282–1311. 10.3758/s13423-016-1220-5 28108977

[B35] Vakil E. Lifshitz-Zehavi H. (2012). Solving the Raven Progressive Matrices by adults with intellectual disability with/without down syndrome: different cognitive patterns as indicated by eye-movements. *Res. Dev. Disabil.* 33 645–654. 10.1016/j.ridd.2011.11.009 22186631

[B36] Vendetti M. S. Starr A. Johnson E. L. Modavi K. Bunge S. A. (2017). Eye movements reveal optimal strategies for analogical reasoning. *Front. Psychol.* 8:932. 10.3389/fpsyg.2017.00932 28626443 PMC5454047

[B37] Vigneau F. Caissie A. F. Bors D. A. (2006). Eye- movement analysis demonstrates strategic influence on intelligence. *Intelligence* 34 261–272. 10.1016/j.intell.2005.11.003

